# Tailored individual Yoga practice improves sleep quality, fatigue, anxiety, and depression in chronic insomnia disorder

**DOI:** 10.1186/s12888-022-03936-w

**Published:** 2022-04-14

**Authors:** Denis Turmel, Sarah Carlier, Anne Violette Bruyneel, Marie Bruyneel

**Affiliations:** 1 Viniyoga, Brussels, Belgium; 2 Department of Pulmonary Medicine, CHU Saint-Pierre, Brussels, Belgium and Université Libre de Bruxelles (ULB), Brussels, Belgium; 3 Geneva School of Health Sciences, HES-SO University of Applied Sciences and Arts Western Switzerland, Geneva, Switzerland

**Keywords:** Anxiety, Chronic insomnia, Sleep, Viniyoga, Yoga, Yoga Cikitsā

## Abstract

**Background:**

Chronic insomnia disorder (CI) is a prevalent sleep disorder that can lead to disturbed daytime functioning and is closely associated with anxiety and depression. First-choice treatment is cognitive behavioral therapy (CBT-I). Other mind–body interventions, such as Tai-chi and Yoga, have demonstrated subjective improvements in sleep quality. The purpose of this study was to assess the efficacy of Yoga for improvement of subjective and objective sleep quality as well as measures of anxiety, depression, sleepiness, and fatigue in patients with CI.

**Methods:**

Adults with CI were prospectively included in this single group pre-post study. Baseline assessments included home polysomnography (PSG), 7-day actigraphy, and questionnaires (Pittsburgh Sleep Quality Index questionnaire (PSQI), Hospital Anxiety Depression scale (HADS), Epworth Sleepiness Scale (ESS), Pichot fatigue scale (PS)). Patients practiced Viniyoga, an individualised Yoga practice with daily self-administered exercises, for 14 weeks. Assessments were repeated at the end of Yoga practice.

**Results:**

Twenty-one patients completed the study. Objective sleep measurements revealed no change in PSG parameters after Yoga practice, but a decrease in arousals on actigraphy (*p* < 0.001). Subjective symptoms improved for all questionnaires (PSQI, *p* < 0.001; HAD-A, *p* = 0.020, HAD-D, *p* = 0.001, ESS, *p* = 0.041, PS, *p* = 0.010). In univariate correlations, decrease in PSQI was associated with increase in sleep stage N3 (*p* < 0.001) on PSG.

**Conclusions:**

We have demonstrated a positive impact of individualized Yoga practice on subjective parameters related to sleep and daytime symptoms in CI, resulting in fewer arousals on actigraphy. Yoga could be proposed as a potentially useful alternative to CBT-I in CI, as it is easy to practice autonomously over the long-term. However, given the design of the present study, future prospective controlled studies should first confirm our results.

**Trial registration:**

ClinicalTrials.gov identifier: NCT03314441, date of registration: 19/10/2017.

**Supplementary Information:**

The online version contains supplementary material available at 10.1186/s12888-022-03936-w.

## Background

Sleep disorders are very common in the general population. One of the most prevalent disorders is chronic insomnia (CI) with a prevalence of about 10%-22% [1, 2]. This prevalence is increasing and rising rates of insomnia have been attributed to changes in lifestyle and environmental factors with, for example, less synchrony with natural wake-sleep rhythms [3]. Insomnia disorder occurs when, despite adequate opportunity for sleep, patients complain of difficulties falling asleep, maintaining sleep, or experiencing sleep as non-restorative and of poor quality, and these problems occur at least three times per week for at least one month.

Sleep complaints in insomnia are accompanied by disturbed daytime functioning. Patients report fatigue and mood disturbances (International Classification of Sleep Disorders (ICSD) -3 [4]). Insomnia is more common in women, especially following menopause, and in older adults. In addition, this disorder can have long-term negative health consequences (i.e. depression, hypertension, immune dysfunction, poor glycemic control) [5]. Insomnia is closely associated with anxiety and depression [6] and insomnia patients suffer from different forms of hyperarousal (cognitive, somatic, or cortical). Due to sleep misperception, people with insomnia have a tendency to overestimate the size of their sleep problem when compared to objective assessment by polysomnography (PSG). Negative conditioning also contributes to the chronic nature of the problem as patients pay too much attention to their sleep and react with maladaptive coping strategies.

Cognitive behavioral treatment for insomnia (CBT-I) is the evidence-based first choice treatment for chronic insomnia and has been endorsed by several North American and European scientific societies [7]. CBT-I is a non-pharmacologic psychotherapeutic intervention that combines sleep restriction, stimulus control, sleep hygiene, education, and cognitive therapy. Growing evidence supports the use of this technique for chronic insomnia [8] as it avoids pharmacologic side-effects and provides sustained long-term efficacy [5, 9]. Indeed, despite their limited efficacy, pharmaceutical hypnotics are often used [10] but should be restricted to acute insomnia treatment. Disappointingly, the availability of CBT-I remains poor [8]. More recently, Mind–Body interventions (MBI), including Tai-chi and Yoga, have shown subjective improvement on sleep quality, sleep efficiency (SE), sleep duration, and sleep latency in randomized controlled trials (RCT) [11]. Yoga practices (breathing and relaxation techniques) can help improve emotional health in anxiety, depression [12], and stress, a key contributor to chronic insomnia [13].

The aim of the present study was to assess the efficacy of Yoga for alleviating chronic insomnia, in both subjective and objective sleep quality, and also on anxiety, depression, sleepiness, and fatigue.

## Methods

### Study design

This was a prospective single group pre-post study performed in the sleep unit of the Saint-Pierre University Hospital in Brussels, Belgium (tertiary referral hospital). Subjective and objective sleep quality, anxiety, depression, sleepiness, and fatigue were assessed at baseline and at the end of the Yoga program.

### Patients

All newly diagnosed adults with chronic insomnia defined by ICSD-3 criteria [4] were included on voluntary basis if they agreed to follow the Yoga program rather than the reference therapy, CBT-I, as first-line treatment. MB included the patient. Exclusion criteria were: known sleep disorder other than insomnia, hypnotics intake, regular Yoga practice, pregnancy, alcohol, or drug abuse.

All included patients provided written informed consent to participate in the study. The study protocol was approved by the Saint-Pierre University Hospital ethics committee (B076201733641). All procedures performed in studies involving human participants were in accordance with the ethical standards of the institutional and/or national research committee and with the 1964 Helsinki declaration and its later amendments or comparable ethical standards. The trial was registered prior to the start of the study: ClinicalTrials.gov identifier: NCT03314441, date of registration: 19/10/2017.

### Baseline measurements

The measurements (baseline-end of study) were performed by SC and by the sleep lab technicians.

#### Polysomnography (PSG)

One complete home-PSG was performed with a portable device (DREAM F, Medatec, Belgium), that allows the recording of thoracic and abdominal movements using piezoelectric sensors, airflow by nasal prongs, pulse oximetry, five electroencephalogram channels, right and left electrooculograms, submental electromyogram (EMG), one anterior tibialis EMG, and electrocardiogram. Tracheal sounds were recorded via a microphone, and body position by a built-in position sensor (mercury gauge) with four different levels. The sleep technician fitted the material around 20:00 at home, and patients were asked to go to bed at their usual time, to note carefully their lights-out time and to remove oxymetry probe when they wake up in the morning. The patient has to remove the monitor himself in the morning and to return it to the sleep lab.

The PSGs were scored manually by one scorer (MB), according to American Academy of Sleep Medicine scoring rules [14].

In normal adults aged 41–50, sleep onset latency (SOL) is about 14–17 min, rapid eye movement (REM) latency is 69–78 min while mean sleep duration is 404–416 min and sleep efficiency (SE) is 84–87%. Sleep stage N3 accounts for 2%-8% of sleep and REM sleep for 20%-22%. Arousal index is 15–16/hour of sleep [15].

#### Actigraphy

The Bodymedia SenseWear Pro Armband® activity monitor was used to record total sleep time (TST), time in bed (TIB), SE, naps, and number of nighttime arousals [16]. Activity was assessed through measurement of total estimated energy expenditure and mean metabolic equivalent of tasks (METs) and the number of steps walked/day. The monitor was attached to an adjustable Velcro armband worn on the non-dominant upper arm for 7 days. Subjects were instructed to wear it around-the-clock with the exception of activities (i.e. showering) that could get the activity monitor wet. Normal daily physical activity for young adults is 6300–7840 steps and 37–51 min of time spent > 3METs [17].

### Questionnaires

#### Pittsburgh Sleep Quality Index questionnaire (PSQI)

Sleep quality and quantity were assessed subjectively using the PSQI [18]. The PSQI includes seven components of subjective sleep: sleep quality, SOL, sleep duration, SE, sleep disturbance, the use of sleep medications, and daytime dysfunction. The overall score ranges from 0 to 21, with higher scores indicating poor quality sleep and scores below 5 considered to be high-quality sleep.

#### Hospital Anxiety Depression scale (HADS)

HADS was used to identify depression and anxiety among patients in this non-psychiatric care setting [19]. This questionnaire includes an anxiety subscale (HADS-A) and a depression subscale (HADS-D) with 14 mixed items. Each item is rated on a four-point scale (0–3), yielding maximum scores of 21 for anxiety and depression. Scores > 11 on either subscale indicate significant psychological comorbidity, a score of 8–10 is borderline, and a score of 7 or below is considered to be normal.

#### Epworth Sleepiness Scale (ESS)

ESS was used to assess daytime sleepiness. A score ≥ 10 indicates excessive daytime sleepiness.

#### Pichot fatigue scale (PS)

This scale includes 8 items. Each item is rated on a five-point scale (0–4). A score ≥ 22 indicates excessive fatigue.

### Yoga practice program

Yoga is a non-invasive mind–body intervention. Unlike studies published thus far, which apply the same Yoga practice to each patient, the type of Yoga practiced in this study is an individualised Yoga form called Viniyoga. Viniyoga can be translated into correct adaptation of the tools of Yoga, and is often used for therapeutic applications or Yoga Cikitsā [20]. Viniyoga was developed by T Krishnamacharya (1888–1989), who is considered as the father of modern Yoga. It was then made more broadly known to the West by his son, TKV Desikachar (1938–2016), who invented the yoga stick figures [21]. Stick figure guidelines were published in 1981 [22]. Until then, Yoga was essentially an oral tradition. In this sense, the stick figures, which represent the Yoga practices in simple graphic forms, were revolutionary. They became instrumental to share the practice with patients. They provide clear guidelines to help patients memorise the practice, which is particularly useful when they are on their own.

- beginning of the program
  The patients were invited by DT to practice individual tailored Viniyoga daily over a 14-week period, following four consultations which took place face-to-face at the teacher’s Yoga studio. As soon as tests were completed at the hospital, the Yoga teacher (DT) contacted the patient. An email was sent explaining the four core rules of the module: 1. Face-to-face sessions during weekends. 2. Inform the teacher 48 h in advance if there is an agenda conflict. 3. Regular practice throughout the module (daily). 4. Commitment to practice until the end of the fourteen-week module. In this introductory email, the proposed plan was already set up: face-to-face consultations on Week 1, Week 3, Week 8 and Week 14, so that each patient has sufficient time to arrange for those meetings.
- Yoga sessions details
  Week 1 (1h30): a dialogue was initiated to gain an in-depth understanding of the patient’s insomnia issue. The practice was designed via a co-creation process with the patient, in order to individualise the practice according to patient’s characteristics. Week 3 (1h): the practice was reviewed and adjusted based on the patient’s feedback. Week 8 (1h): the practice was discussed again and fine-tuned. Week 14 (1h30): the overall Yoga program was debriefed and final recommendations were provided to the patient for continuing the practice of Yoga independently.
  Thus, an assessment was done during each face-to-face session and patients were also allowed to contact the Yoga teacher to clarify their practice, when required.
  To provide additional support, an A4 sheet describing the practice in the form of the stick-figures described earlier was given to the patient at the end of each consultation. If necessary, guided meditations were recorded and provided to the patients by e-mail. The ultimate goal, however, was that patients would become empowered to continue practicing on their own by the end of the 14-week module.
- Yoga practice content
  In the TKV Desikachar’s translation of the Yoga Sūtra of Pantanjali, Yoga is defined as “the ability to direct the mind exclusively towards an object and sustain that direction without any distractions” [21]. This is the essence of how Yoga was taught in this study. Several tools can be used in yoga, but a fundamental tool used in all practices was to help patients reconnect with their breathing. Indeed Yoga establishes a direct connection between one’s breathing pattern and one’s state of mind. Learning to focus and gradually to better control the breathing helps to better control the mind and to reduce the flow of thoughts which often prevents patients from falling asleep or to fall back to sleep.

The essence of Viniyoga is to tune the practice to each patient. This is essential for patients to adhere to the practice. The individualised consultations took into account the context of patients in a very broad sense to best adapt the practice to patient’s needs: the nature of sleeping issue (difficulty falling asleep, difficulty returning to sleep, early wake up, lack of energy during the day, etc.), the time available to practice, the patient’s personal context (life surroundings, parent with children, elderly), lifestyle (eating and drinking habits, nature of activities prior to sleep). The Yoga teacher stressed the importance of regular practice (i.e. daily), rather than its length. Some exercises were also given during the day to help embed new ways of focussing. The nature of the exercises was such that patients were able to practice them almost anywhere: in their bedroom, just before going to sleep, in bed if the night was interrupted, in the morning while waking up, at work at their desk, in public transport, or in public places, in order to ensure frequent and regular practice. All exercises provided were actually practiced with the patient, until the patient was sufficiently autonomous to practice on his own at home. This was also essential to help patient practice daily.

Unlike sport, where it is usual to feel pain during or after the exercise, Viniyoga emphasizes that patients should learn to respect their body. There was a step-by-step progression (called Vinyasa Krama in Yoga) week after week, but also within the practice. This progression ensured that patients could practice Yoga safely and within their own limits. Practices could take from five minutes, two to three times a day, up to 30 min.

Yoga exercises included a variety of traditional Yoga tools such as postures (Āsana), breathing practices (Prānāyāma), visualisations (Bhāvanā), coordination of breathing with touching one’s own body (Nyāsa), meditation (Dhyāna), and sometimes the use of mantra. Yoga often involves repeating the same exercise a specific amount of times, which requires focus. Most patients have difficulty counting in their heads while focusing on breathing and movement. To make it easier, patients are taught the simple Yoga finger counting system where the thumb is used as a pointer touching each phalange one after the other, as described in Appendix. All these tools can be combined to best suit the patient’s needs and preferences. Examples of such exercises are provided as Appendix. Though many of these exercises are broadly applicable, specifics of these practices might not necessarily be appropriate to all patients.

### End of study measurements

Three months later, at the end of Yoga practice, the baseline measurements were repeated.

### Statistical analyses

Statistical analyses were performed by AVB. They include descriptive statistics. As all data didn’t follow a normal distribution, the median and quartiles were reported. The quartiles divided the distribution into four classes of equal size and were noted Q1 (25%), Median (50%) and Q3 (75%).

The median and Q1 (25%), Q3 (75%) were reported for polysomnographic, actimetry variables, and questionnaires for baseline and post treatment values. The normality of the data was tested by the Shapiro–Wilk test. Comparisons between baseline and post treatment values were evaluated by paired t-test when the data had a normal distribution. If the data were non-normally distributed variables, a non-parametric Wilcoxon test was applied.

In order to evaluate the relationship between the evolution of the variables, we calculated the delta (post-treatment value—baseline value) for each subject for polysomnographic, actimetry, and questionnaire variables. Spearman correlations were then applied to evaluate the relationship between:

1. questionnaire deltas and PSG deltas
2. number of naps deltas and PSG deltas
3. HAD A score deltas and step/time > 3 METs deltas

A value of *p* < 0.05 was considered significant.

All analyses were performed using python (version 3.8.3) with the statistics libraries Statsmodels (version 0.12.0) and SciPy (version 1.5.2). These packages are released under the open source – OSI (Open Source Initiative) approved—modified BSD (Berkeley Software Distribution) (3-clause) license.

## Results

Between November, 2017 and December, 2020, 45 patients were screened and 23 included (9 males). Reasons for non-inclusion included a long delay between diagnosis and Yoga courses (= 12), loss of motivation (*n* = 8), and new onset of medical problems (*n* = 2). Each patient was a volunteer to experiment Yoga to improve his/her sleep, and 21/23 completed the program. Two stopped on the way for the following reasons: one following the birth of his baby and one who suffered a severe health issue. Mean symptom duration was 13 months. In patients with a history of hypnotic use, the mean duration of treatment was 74 months (range 1–240). The last intake occurred at the time of inclusion in the study (consultation with MB) in 4/21 patients. For the remaining 17 patients, the last intake was on average 50 months earlier (range 1–180).

Insomnia characteristics are reported in Table 1.

**Table 1:** Demographic and clinical characteristics of the study population

Age (years), median (min–max)	45 (28–58)
**• Men, *n* = 9**	46 (30–57)
**• Women, *n* = 12**	41 (28–58)
Insomnia symptoms	
difficulty falling asleep	86%
difficulty maintaining sleep	67%
early morning awakening	33%
non-refreshing sleep	33%
History of hypnotics intake	
benzodiazepines	29%
Z-drugs	19%
trazodone	19%
mirtazapine	9%
melatonin	9%
NONE	48%

At baseline, sleep structure on both PSG and actigraphy was within the normal range for all patients, while all complained of poor sleep. A total of 13/21 (62%) exhibited borderline-to-overt anxiety (HADS-A ≥ 8).

Among the 21 patients who followed the yoga module to completion (four face-to-face consultations, 14 weeks of daily practice), none reported any adverse side effect.

**Fig. 1:**
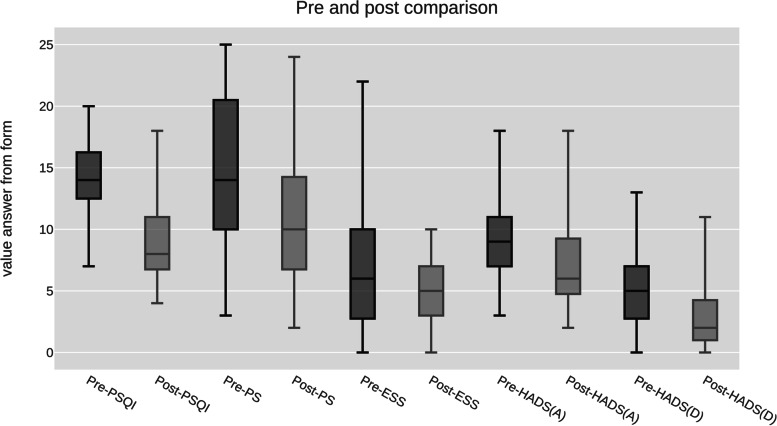
Pre and post-Yoga program comparison of questionnaire scores. All the pre-post values comparisons are significant, with a *p* < 0.005. PSQI: Pittsburgh Sleep Quality Index, PS: Pichot fatigue scale, ESS: Epworth Sleepiness Scale, HADS: Hospital Anxiety Depression scale, (A): anxiety, (D): depression

At the end of the Yoga program, we observed an improvement in all subjective parameters but no change in PSG and actigraphy parameters, except for the number of arousals measured by actigraphy. These results are summarized in Table 2 and Fig. 1.

**Table 2:** Results of baseline and post-Yoga practice sleep, activity, and symptom assessments

Variables Median (Q1,Q3)		Baseline measurements	Post-3 months Yoga practice measurements	*p* value
Polysomnography	total sleep time (min)	402(336–456)	408(352–440)	0.68
	sleep efficiency (%)	82.2(71.4–91.3)	86.9(80.6–90.2)	0.25
	sleep onset latency (min)	18(12–34)	15(12–33)	0.30
	REM latency (min)	98(67–129)	111(76.5–142)	0.29
	Stage N1(%)	3.1(1.3–4.1)	2.5(1.7–3.4)	0.22
	Stage N2(%)	51.3(39.4–59.2)	48(45–55.5)	0.90
	Stage N3(%)	24.2(18.2–28.7)	24.6(18.7–31.3)	0.29
	Stage REM(%)	22.4(17.9–26.6)	20.5(15.5–23.8)	0.21
	Arousal index (/h sleep)	10.4(6.1–12.4)	8.2(6.8–10.5)	0.64
Actigraphy	time in bed (min)	532(499–551)	518(465–536)	0.13
	total sleep time (min)	421(375–456)	434(375–473)	0.82
	sleep efficiency (%)	80.2(77.7–84.9)	85.2(78.7–89.7)	0.11
	Arousals (n)	10.8(7–12.7)	7.9(6.7–10.6)	< *0.01*
	naps (n)	0.3(0.17–0.5)	0.4(0–0.7)	0.20
	steps walked/day (n)	7406(5657–8853)	7060(6383–9717)	0.23
	Time spent > 3METs/day (min)	118(63–152.8)	85(64–130)	0.15
Questionnaires	Pittsburgh Sleep Quality Index	14(13–16)	8(7–11)	< *0.001*
	HADS-A	9(7–11)	6(5–9)	*0.02*
	HADS-D	5(3–7)	2(1–4)	< *0.001*
	Epworth Sleepiness Scale	6(3–9)	5(3–7)	*0.04*
	Pichot fatigue scale	14(11–20)	10(7–14)	*0.01*

*HADS* Hospital Anxiety Depression scale, *REM* rapid eye movement, *METs* metabolic equivalent of tasks

In univariate correlations, decrease in PSQI was associated with decrease in sleep N2 (*p* < 0.001) and increase in sleep N3 (*p* < 0.001) on PSG. Decrease in fatigue (PS) was correlated with fewer arousals (*p* = 0.05) and more REM sleep (*p* = 0.04) on PSG.

## Discussion

In the present study, we have demonstrated a positive impact of individually-tailored Yoga practice on subjective parameters related to sleep quality, sleepiness, fatigue, anxiety, and depression in insomnia patients. The only objective improvement of sleep structure was observed with actigraphy, with a significant reduction in arousals measured over a 7-day period.

The diagnosis of insomnia, according to ICSD-3 classification, is purely subjective and based on daytime and nighttime patient complaints [3]. No quantitative criteria for sleep-onset latency, sleep duration, or the frequency/duration of nocturnal awakenings are required to define the disorder. PSG is not considered helpful for diagnosis of insomnia, as it does not correlate with the perception of the patients [8]. Sleep misperception is a common trait of the disorder, and is characterized by a mismatch between patient’s sleep appraisals and objective measurements of sleep [23]. Insomnia can thus be considered to be a subjective disorder, more frequently present in patients with specific personality traits (neuroticism, maladaptive perfectionism) [8].

This particularity of insomnia disorder can explain that we did not observe improvement of sleep quality on PSG. Indeed, despite insomnia complaints, measures of objective sleep quality were already in the normal range at baseline. However, we did observe that increases in stage N3 sleep were associated with a decrease in PSQI score, indicating that when the sleep quality improves, insomnia complaints are reduced. Similar findings have been observed with transcranial magnetic simulation [24].

Insomnia is associated with increased psychological symptomatology, arousals, perceived stress, and poor quality of life [25]. Physiopathology emphasizes the role of precipitating and perpetuating factors [26]. According to these characteristics, a holistic approach is necessary to manage these patients. This is also the cause of the limited impact of pharmacotherapy.

Mind–body interventions (MBIs) combine several techniques with the purpose of enhancing the capacity of the mind to influence bodily functions and symptoms [25]. It is hypothesized to act by restoring sympathetic/parasympathetic balance and decreasing arousals [27]. Cognitive restructuring is also an essential element. MBIs that have been tried for insomnia treatment include Mindfulness (stress reduction techniques and cognitive elements), hypnotherapy, Tai-Chi (slow moving meditation with diaphragmatic breathing and relaxation), CBT-I, relaxation, and Yoga [5, 25, 27, 28].

Yoga is an ancient philosophy, originating in India, that aims to bring harmony between mind and body. Mindfulness, is an essential component of Yoga. In this sense, Āsana and Prānāyāma can be seen as tools for learning to focus the mind on the body and on the breathing, respectively. Yoga has been adapted to be practiced around the world on various ways [29]. Different possible mechanisms of action could explain its positive effects in several disorders. The exercise component of Yoga has been shown to be able to increase thalamic GABA, to modulate serotoninergic and noradrenergic systems and to release opioids. The mindfulness component influences the hypothalamic- pituitary-adrenal system and decreases cortisol levels. Finally, breathing control could lead to a recalibration of sympathetic nervous system via vagal stimulation. Yoga has also been shown to increase melatonin levels and improve immune response [12, 29–31]. A recent meta-analysis based on 3 RCTs on the effects of Yoga for anxiety concluded that Yoga might be an effective and safe intervention for individuals with elevated levels of anxiety [12]. The reduction of anxiety complaints, present in the majority of the study population, confirm Yoga’s effectiveness on stress reduction, with a potential direct impact on insomnia. Increase in melatonin levels can add to insomnia improvement [32].

In the present study, we show for the first time that Yoga improves subjective sleep quality and daytime functioning that are related to significant objective changes in sleep structure on actigraphy, that demonstrated a reduction of arousals. Previous reports that studied Yoga’s effectiveness in insomnia were mainly based on subjective measurements. Indeed, studies have shown subjective improvements in sleep quality and quantity after 14 days in 20 adults suffering from sleep-onset or sleep-maintenance insomnia [33], a positive effective on sleep onset latency and sleep duration after 6 months of Yoga practice in a geriatric population, compared to Ayurveda or no intervention [34], and a lower stress score in 20 insomnia male patients compared to 20 controls after 8 weeks [35]. A recent meta-analysis, including 1832 participants from RCTs, studied the effects of Yoga in women suffering from insomnia, and concluded that subjective sleep quality improvement occurs but not in women suffering from breast cancer and in peri/post-menopausal women [29]. The only study reporting PSG records refers to post-menopausal women. Afonso et al. compared Yoga with passive stretching and no intervention, showing a reduction of subjective insomnia severity with Yoga. PSG details are not reported but are described as not different between groups [36]. In two other studies, actigraphy was used to measure sleep, and no objective effect was observed with Yoga. The studied population was quite different from our population of insomnia patients without comorbidities. In the first study, women suffering from breast cancer exhibited a subjective modest benefit on PSQI while practicing twice a week for 3–6 months, and in the second, having included post-menopausal women, no effect of Yoga was observed [37, 38]. Indeed, sleep disturbances around menopause seem to be a distinct pattern of insomnia, where somatic conditions can occur (vasomotor symptoms, obstructive sleep apnea) that are less likely to respond to Yoga or CBT-I [39]. The positive impact of Yoga in the present study is probably also related to the personalized approach of Viniyoga and the use of stick figures. All our patients, men and women, were able to practice, even with no prior experience and despite a wide age range (28 to 58 years old). In fact, Yoga is not about acrobatic asana and can be practiced by anyone, supporting to its potential for broad use as an alternative to CBT-I in insomnia patients, and allowing long-term autonomous practice.

### Limitations and strengths

The tailored individual Yoga approach used in this study was certainly an interesting dimension for personalized care with an important positive psychological dimension. However, these results must be put into perspective given the design and small size of the study. Indeed, this single-group pre-post study did not have a control group or comparator to confirm the efficacy of yoga treatment. Moreover, a placebo effect cannot be excluded, as it can lead to objective and subjective changes in sleep.

In conclusion, we have shown, for the first time, a positive impact of individually-tailored Yoga practice on subjective parameters related to sleep quality, sleepiness, fatigue, anxiety, and depression in insomnia patients. These positive changes were related to objectively fewer arousals on actigraphy. Increase in sleep stage N3 on PSG also correlated directly with better sleep quality. Yoga could be proposed as a potentially useful alternative to CBT-I in chronic insomnia, as it is easy to practice autonomously over the long-term. However, given the design of the present study, future prospective controlled or comparative studies should first confirm our results. Other aspects of Yoga need also to be further investigated: it would be useful to design studies focusing on the pathophysiological aspect of yoga to better understand its effectiveness and perhaps isolate the most effective aspect of the practice (physical exercise, breathing control, mindfulness).

## Supplementary Information

**Additonal file 1.**

## Data Availability

The datasets used and analyzed during the current study are available from the corresponding author on reasonable request.

## Abbreviations

CBT-I: Cognitive behavioral treatment for insomnia
ESS: Epworth Sleepiness Scale
HADS: Hospital Anxiety Depression scale
MBI: Mind–body intervention
MET: Metabolic equivalent of tasks
PSG: Polysomnography
PSQI: Pittsburgh Sleep Quality Index questionnaire
PS: Pichot Fatigue Scale
SE: Sleep efficiency
TST: Total sleep time
TIB: Time in bed
RCT: Randomized clinical trial

## References

[1] Roth T (2007). Insomnia: definition, prevalence, etiology, and consequences. J Clin Sleep Med.

[2] Roth T, Coulouvrat C, Hajak G, Lakoma MD, Sampson NA, Shahly V , Shillington AC, Stephenson JJ, Walsh JK, Kessler RC. Prevalence and perceived health associated with insomnia based on DSM-IV-TR; International Statistical Classification of Diseases and Related Health Problems, Tenth Revision; and Research Diagnostic Criteria/International Classification of Sleep Disorders, Second Edition criteria: results from the America Insomnia Survey, Biol Psychiatry 2011;10.1016/j.biopsych.2010.10.023 10.1016/j.biopsych.2010.10.023 21195389

[3] Kennedy SL (2014). Yoga as the “next wave” of therapeutic modalities for treatment of insomnia. Int J Yoga Therap.

[4] Sateia MJ. International classification of sleep disorders-third edition: highlights and modifications. Chest. 2014;146(5):1387–94. 10.1378/chest.14-0970. 10.1378/chest.14-0970 25367475

[5] Miller MA, Renn BN, Chu F, Torrence N (2019). Sleepless in the hospital: A systematic review of non-pharmacological sleep interventions. Gen Hosp Psychiatry.

[6] Taylor DJ, Lichstein KL, Durrence HH, Reidel BW, Bush AJ (2005). Epidemiology of insomnia, depression, and anxiety. Sleep.

[7] Yu JS, Kuhn E, Miller KE, Taylor K (2019). Smartphone apps for insomnia: examining existing apps’ usability and adherence to evidence-based principles for insomnia management. Transl Behav Med.

[8] Riemann D, Baglioni C, Bassetti C, Bjorvatn B, Dolenc Groselj L, Ellis JG (2017). European guideline for the diagnosis and treatment of insomnia. J Sleep Res.

[9] Geiger-Brown JM, Rogers VE, Liu W, Ludeman EM, Downton KD, Diaz-Abad M (2015). Cognitive behavioral therapy in persons with comorbid insomnia: A meta-analysis. Sleep Med Rev.

[10] Tariq SH, Pulisetty S (2008). Pharmacotherapy for insomnia. Clin Geriatr Med.

[11] Sarris J, Byrne GJ (2011). A systematic review of insomnia and complementary medicine. Sleep Med Rev.

[12] Cramer H, Lauche R, Anheyer D, Pilkington K, de Manincor M, Dobos G (2018). Yoga for anxiety: A systematic review and meta-analysis of randomized controlled trials. Depress Anxiety.

[13] Agrawal G (2013). A Review of the Psychological Benefits of Yoga. Int J Yoga Allied Sci.

[14] Berry RB, Budhiraja R, Gottlieb DJ, Gozal D, Iber C, Kapur VK (2012). American Academy of Sleep Medicine, Rules for Scoring Respiratory Events in Sleep: Update of the 2007 AASM Manual for the Scoring of Sleep and Associated Events. J Clin Sleep Med.

[15] Hertenstein E, Gabryelska A, Spiegelhalder K, Nissen C, Johann AF, Umarova R (2018). Reference Data for Polysomnography-Measured and Subjective Sleep in Healthy Adults. J Clin Sleep Med.

[16] Withrow DJ, Roth T, Koshorek G, Roehrs T (2019). Relation between ambulatory actigraphy and laboratory polysomnography in insomnia practice and research. J Sleep Res.

[17] Dalibalta S, Majdalawieh A, Yousef S, Gusbi M, Wilson JJ, Tully MA (2021). Objectively quantified physical activity and sedentary behaviour in a young UAE population. BMJ Open Sport Exerc Med.

[18] Buysse DJ, Reynolds CF, Monk TH, Berman SR, Kupfer DJ (1989). The Pittsburgh Sleep Quality Index: a new instrument for psychiatric practice and research. Psychiatry Res.

[19] Zigmond AS, Snaith RP (1983). The Hospital Anxiety and Depression scale. Acta Psychiatr Scand.

[20] Chandrasekaran N (2012). Principles and Practices of Yoga Therapy Vhf Publications Chennai.

[21] Desikachar TKV (1999). The Heart of Yoga, Inner Traditions Ed.

[22] Smith MJN (2007). An Illustrated Guide to Āsanas and Prānāyāma, Krishnamacharya Yoga Mandiram Ed.

[23] Edinger JD, Krystal AD (2003). Subtyping primary insomnia: is sleep state misperception a distinct clinical entity?. Sleep Med Rev.

[24] Sun N, He Y, Wang Z, Zou W, Liu X (2021). The effect of repetitive transcranial magnetic stimulation for insomnia: a systematic review and meta-analysis. Sleep Med.

[25] Kozasa EH, Hachul H, Monson C, Pinto L, Garcia MC, de AraújoMoraes Mello LE (2010). Mind-body interventions for the treatment of insomnia: a review. Braz J Psychiatry.

[26] Ellis JG, Perlis ML, Espie CA, Grandner MA, Bastien CH, Barclay NL, et al. The Natural History of Insomnia: Predisposing, precipitating, coping and perpetuating factors over the early developmental course of insomnia. Sleep 2021; 10.1093/sleep/zsab095. Epub ahead of print. 10.1093/sleep/zsab095 PMC8826168 33849074

[27] Irwin MR, Olmstead R, Motivala SJ (2008). Improving sleep quality in older adults with moderate sleep complaints: A randomized controlled trial of Tai Chi Chih. Sleep.

[28] Rusch HL, Rosario M, Levison LM, Olivera A, Livingston WS, Wu T (2019). The effect of mindfulness meditation on sleep quality: a systematic review and meta-analysis of randomized controlled trials. Ann N Y Acad Sci.

[29] Wang WL, Chen KH, Pan YC, Yang SN, Chan YY (2020). The effect of Yoga on sleep quality and insomnia in women with sleep problems: a systematic review and meta-analysis. BMC Psychiatry.

[30] Zeichner SB, Zeichner RL, Gogineni K, Shatil S, Ioachimescu O (2017). Cognitive Behavioral Therapy for Insomnia, Mindfulness, and Yoga in Patients With Breast Cancer with Sleep Disturbance: A Literature Review. Breast Cancer (Auckl).

[31] Vadiraja HS, Raghavendra RM, Nagarathna R, Nagendra HR, Rekha M, Vanitha N (2009). Effects of a Yoga program on cortisol rhythm and mood states in early breast cancer patients undergoing adjuvant radiotherapy: a randomized controlled trial. Integr Cancer Ther.

[32] Ferracioli-Oda E, Qawasmi A, Bloch MH (2013). Meta-analysis: melatonin for the treatment of primary sleep disorders. PLoS ONE.

[33] Khalsa SBS (2004). Treatment of chronic insomnia with Yoga: a preliminary study with sleep-wake diaries. Appl Psychophysiol Biofeedback.

[34] Manjunath NK, Telles S (2005). Influence of Yoga and Ayurveda on self-rated sleep in a geriatric population. Indian J Med Res.

[35] Sobana R, Parthasarathy S, Duraisamy, Jaiganesh K, Vadivel S., et al. The effect of Yoga therapy on selected psychological variables among male patients with insomnia. J Clin Diagn Res 2013; 10.7860/jcdr/2012/5056.2669 10.7860/JCDR/2012/5056.2669 PMC3576750 23450219

[36] Afonso RF, Hachul H, Kozasa EH, de Souza OD, Goto V, Rodrigues D (2012). Yoga decreases insomnia in postmenopausal women: a randomized clinical trial. Menopause.

[37] Chaoul A, Milbury K, Spelman A, Basen-Engquist K, Hall MH, Wei Q (2018). Randomized trial of Tibetan yoga in patients with breast cancer undergoing chemotherapy. Cancer.

[38] Buchanan DT, Landis CA, Hohensee C, Guthrie KA, Otte JL, Paudel M (2017). Effects of Yoga and Aerobic Exercise on Actigraphic Sleep Parameters in Menopausal Women with Hot Flashes. J Clin Sleep Med.

[39] Kalmbach DA, Cheng P, Roth T, Sagong C, Drake CL (2020). Objective sleep disturbance is associated with poor response to cognitive and behavioral treatments for insomnia in postmenopausal women. Sleep Med.

