# Supplementary material for: Tailored individual Yoga practice improves sleep quality, fatigue, anxiety, and depression in chronic insomnia disorder
Source: BMC Psychiatry. 2022 Apr 14;22:267. doi: 10.1186/s12888-022-03936-w (PMC9012014; doi:10.1186/s12888-022-03936-w)
Supplement: Supplementary file 1 — Additonal file 1. [file 12888_2022_3936_MOESM1_ESM.zip › Appendix.docx]

**Appendix:** Examples of Viniyoga practices

In each consultations, a sequence of exercises was custom-made to suit each patient, as the nature of the patient's insomnia, the patient's context, the patient's available time, etc varied from patient to patient. Though many of these exercises are broadly applicable, specifics of these practices might not necessarily be -appropriate to or -to the liking of all patients. Illustrative positions or movements (Āsana), breathing practices (Prānāyāma), visualisations (Bhāvanā), coordination of breathing with touching one's own body (Nyāsa), meditation practices (Dhyāna) are given below.

**Yoga finger counting technique (Figure A1)**

In the Yoga finger counting system, the thumb is used as a pointer to count the number of breaths. It touches each phalange one after the other in the spiral sequence shown below.

**Examples of Āsana with props**

Āsana with props is a very simple tool to help patients reconnect with breathing.

**Figure A2**

It describes Āsana practiced with soft balls, in this case practiced twelve times (12X).

- Inhale (IN) : release pressure on balls.
- Exhale (EX) : squeeze balls.

The exercise can be practiced in the evening with a retention after exhale (RaE, relaxing) or in the morning with a retention after inhale (RaI, energizing).

**Figure A3**

A very simple sequence practiced standing up, mostly as an energizing practice for the morning for beginners. An infinite variety of Asana can be developed.

The practice can be first done dynamically (4X to 8X), and thereafter with a retention after inhale of three seconds (4X with 3s RaI).

**Example of Nyāsa (Figure A4)**

Nyāsa is very powerful tool to help patients learn to synchronize simple movements with breathing and to bring focus to different parts of their body.

Nyāsa consists in touching different parts on one's body, in this case staying three breaths (3B) in the position, starting by knees and then thighs, stomach, heart region, and finally palming the eyes. There is an infinite variety of options for that, which can be tuned based on the patient's taste or needs.

The exercise can be done sitting on a chair.

It can also be done by laying down in bed using Bhāvanā ie by visualizing the movements in one's mind.

**Examples of Langhana Prānāyāma**

There is an infinite variety of Prānāyāma styles, fro, very simple to very complex.

An increase in complexity can be used to ensure that patients remain interested in the exercises.

Below Prānāyāma, where a retention after exhale is gradually introduced (Vinyasa Krama) after an observation stage, is generally appropriate before going to bed.

Prānāyāma are classically carried out in seating position. Some patients find it easier to fall asleep when practicing laying down. Also, this Prānāyāma can be done in laying down position if one wakes up in the middle of the night.

The exercise starts by observing breathing (breathing rhythm, amplitude, regularity, difference between length of inhale & exhale) and then starting controlling breathing.

- Prānāyāma convention (a-b-c-d) : a=Inhale – b=retention after inhale – c=exhale – d=retention after exhale. In this case, each breathing sequence is repeated four times (X4).
  1. Observe
  2. (1-0-1-0) x4 🡪 length of inhale = length of exhale
  3. (1-0-1- ½) x4 🡪 exhale longer the inhale
  4. (1-0-1-1) x8 🡪 exhale twice as long as inhale
  5. (1-0-1- ½) x4
  6. (1-0-1-0) x4
  7. Observe

**Examples of Dhyāna**

An infinite variety of meditation practices can be developed to suit each person. They can be practiced in sitting or lying position.

Meditations can be very effective to help patients fall asleep, or falling back to sleep if woken up in the middle of the night. In this case, they can be practiced laying down with eyes closed.

- Meditation No 1: sensing the point of contact of the body with the mattress, starting by the feet, the legs, the lower back, the hand, the arms, the upper back, the back of the head.
- Meditations No 2: drawing the contours of one's body in space.
- Meditation No 3: placing both palms on one's stomach and sensing the movements induced by breathing.

**Examples of simple practices during the day to embed mind-focus**

- Synchronize breathing and walking
- Reconnect with breathing frequently during the day by just observing the breathing pattern without control (breathing rhythm, amplitude, regularity, difference between length of inhale & exhale). Can be done at your desk, during public transportation, etc.
- Meditation focusing on senses, eg whilst sitting on a bench in a park (listen to outside sounds, birds singing – smell the air, the perfume of trees and flowers – feel the wind on your skin, feel the point of contact of your body with the bench – if your eyes are open, look at colors and shapes around)
- Meditation on the present moment (when you listen to someone – when you eat – when you drink – when you wash your hands, etc.
