# Supplementary figures and images for: Tailored individual Yoga practice improves sleep quality, fatigue, anxiety, and depression in chronic insomnia disorder
Source: BMC Psychiatry. 2022 Apr 14;22:267. doi: 10.1186/s12888-022-03936-w (PMC9012014; doi:10.1186/s12888-022-03936-w)

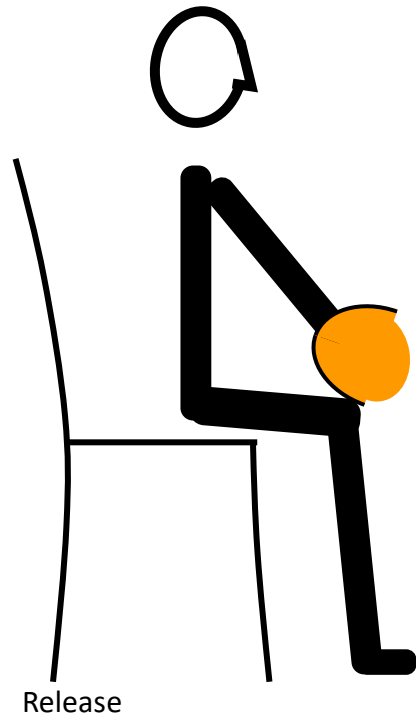

EX

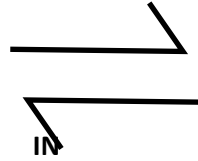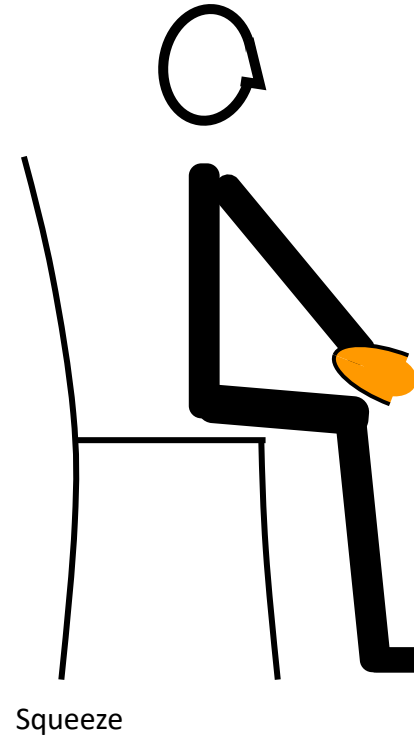

12X

Supplement: Supplementary file 1 — Additonal file 1. [file 12888_2022_3936_MOESM1_ESM.zip › FigA2.pdf]

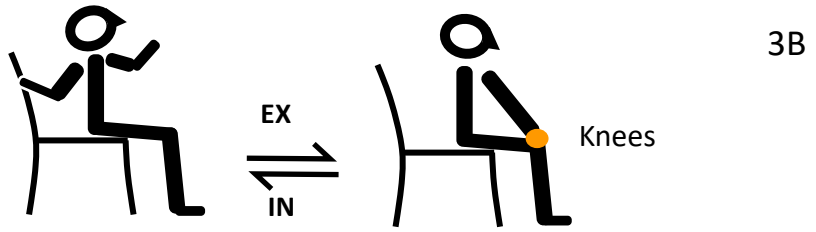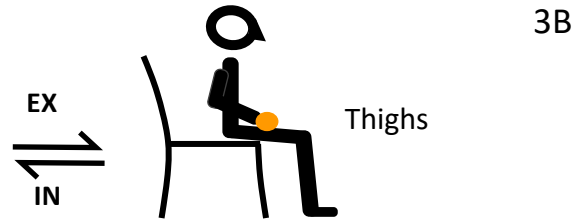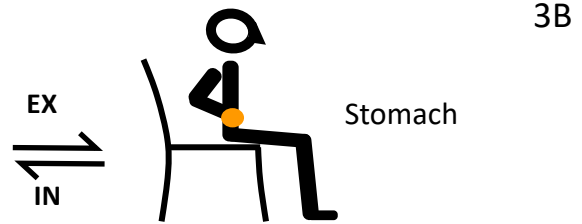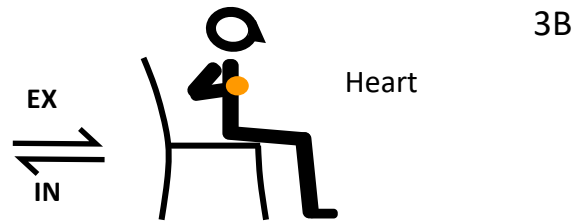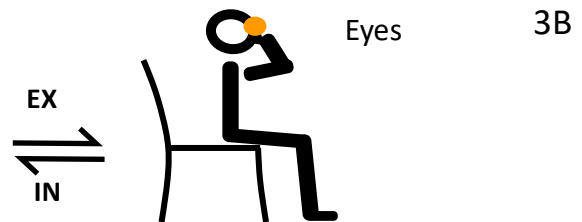

Supplement: Supplementary file 1 — Additonal file 1. [file 12888_2022_3936_MOESM1_ESM.zip › FigA4.pdf]

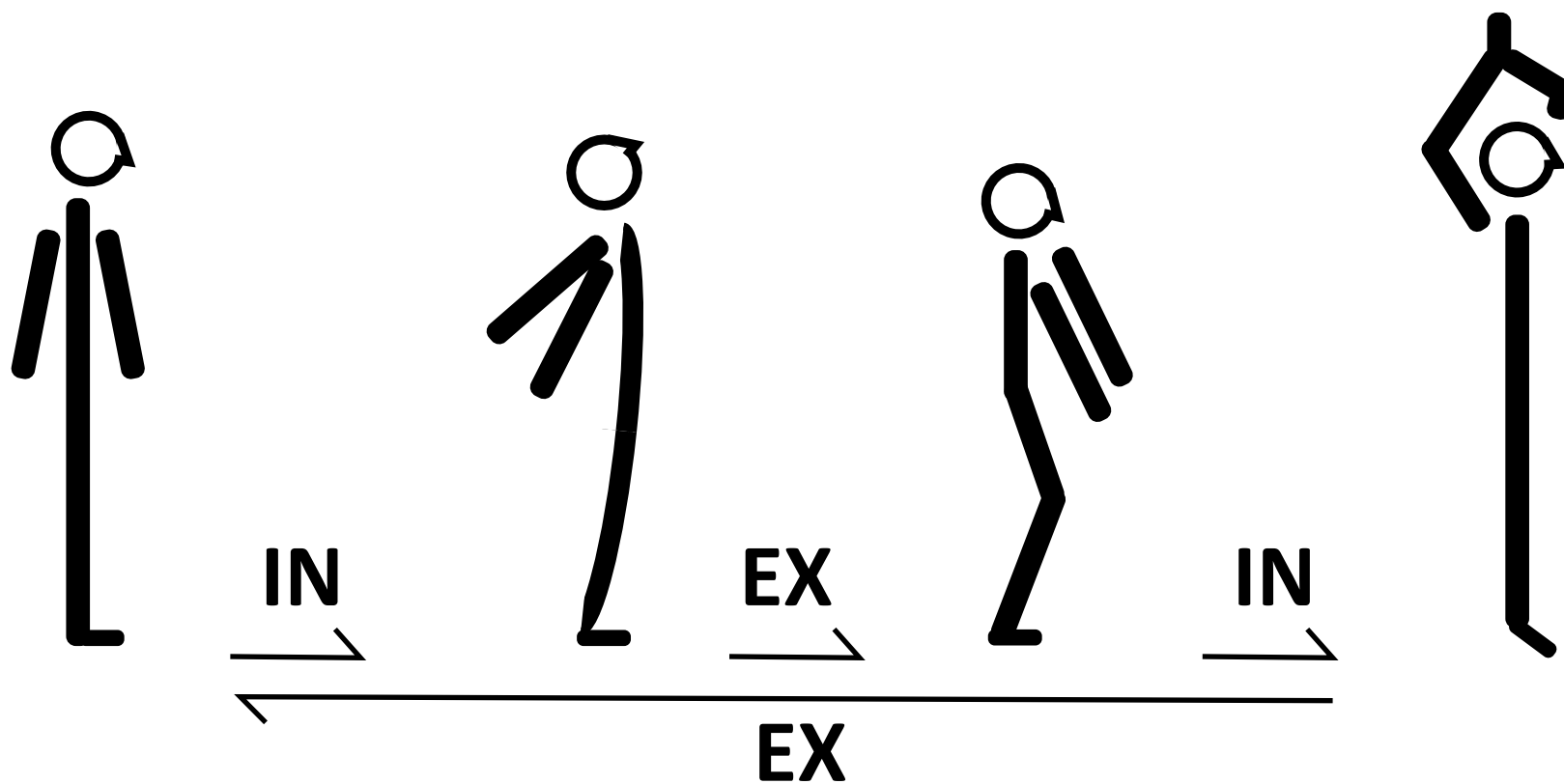

4X to 8X

4X Ral 3s

Supplement: Supplementary file 1 — Additonal file 1. [file 12888_2022_3936_MOESM1_ESM.zip › FigA_3.pdf]

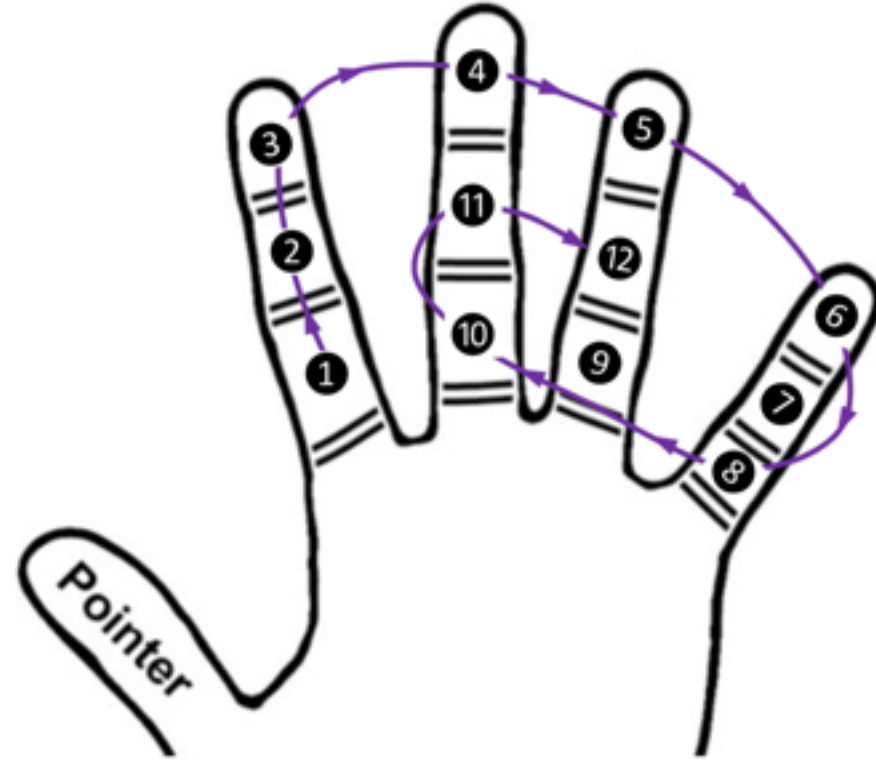

Supplement: Supplementary file 1 — Additonal file 1. [file 12888_2022_3936_MOESM1_ESM.zip › FigureA1.pdf]
